# Supplementary material for: COVID-19: molecular and serological detection methods
Source: PeerJ. 2020 Oct 7;8:e10180. doi: 10.7717/peerj.10180 (PMC7547594; doi:10.7717/peerj.10180)
Supplement: Supplemental Information 1 [file peerj-08-10180-s001.docx]

**Table S1:**

**Primers and probes that have been recommended by other institutions to perform rRT-PCR and detect SARS-CoV-2**

| **Gene rget** | **Description** | **Oligonucleotide Sequence (5’>3’)** | **Label** | **Institution** |
| --- | --- | --- | --- | --- |
| N gene | N-Sarbeco- Forward Primer | CACATTGGCACCCGCAATC | None | Tib-Molbiol, Germany^43^ |
|  | N-Sarbeco- Reverse Primer | GAGGAACGAGAAGAGGCTTG | None |  |
|  | N-Sarbeco- Probe | FAM-ACTTCCTCAAGGAACAACATTGCCA-BBQ | FAM, BBQ |  |
| RdRp gene | RdRp-SARSr- Forward Primer | GTGARATGGTCATGTGTGGCGG | None |  |
|  | RdRp-SARSr- Reverse Primer | CARATGTTAAASACACTATTAGCATA | None |  |
|  | RdRp-SARSr- Probe1 | FAM-CCAGGTGGWACRTCATCMGGTGATGC-BBQ | FAM, BBQ |  |
|  | RdRp-SARSr- Probe2 | FAM-CAGGTGGAACCTCATCAGGAGATGC-BBQ | FAM, BBQ |  |
| E gene | E-Sarbeco- Forward Primer | ACAGGTACGTTAATAGTTAATAGCGT | None |  |
|  | E-Sarbeco- Reverse Primer | ATATTGCAGCAGTACGCACACA | None |  |
|  | E-Sarbeco- Probe | FAM-ACACTAGCCATCCTTACTGCGCTTCG-BBQ | FAM, BBQ |  |
| ORF1ab | ORF1ab- Forward Primer | CCCTGTGGGTTTTACACTTAA | None | CCDC, China^47^ |
|  | ORF1ab- Reverse Primer | ACGATTGTGCATCAGCTGA | None |  |
|  | ORF1ab- Probe | FAM-CCGTCTGCGGTATGTGGAAAGGTTATGG-BHQ1 | FAM, BHQ1 |  |
| E gene | E- Forward Primer | TTCTTGCTTTCGTGGTATTC | None |  |
|  | E- Reverse Primer | CACGTTAACAATATTGCAGC | None |  |
|  | E- Probe | FAM-GTTACACTAGCCATCCTTACTGCGCTTCGA-BHQ1 | FAM, BHQ1 |  |
| N gene | N- Forward Primer | GGGGAACTTCTCCTGCTAGAAT | None |  |
|  | N- Reverse Primer | CAGACATTTTGCTCTCAAGCTG | None |  |
|  | N- Probe | FAM-TTGCTGCTGCTTGACAGATT-BHQ1 | FAM, BHQ1 |  |
| RdRp gene | RdRp- Forward Primer | GGTCATGTGTGGCGGCTC | None |  |
|  | RdRp- Reverse Primer | GCTGTAACAGCTTGACAAATGAAAG | None |  |
|  | RdRp- Probe | FAM-CTATATGTTAAACCAGGTGGAAC-BHQ1 | FAM, BHQ1 |  |
| N gene | 2019-nCOV-N- Forward Primer | AAATTTTGGGGACCAGGAAC | None | NIID,  Japan^48^ |
|  | 2019-nCOV-N- Reverse Primer | TGGCAGCTGTGTAGGTCAAC | None |  |
|  | 2019-nCOV-N- Probe | FAM-ATGTCGCGCATTGGCATGGA-BHQ | FAM, BHQ1 |  |

The Chinese Center for Disease Control and Prevention (CCDC); National Institute of Infectious Diseases (NIID); FAM: 6-carboxyfluorescein; BBQ: blackberry quencher; BHQ-1: Black Hole Quencher-1;. W is A/T; R is G/A; M is A/C; S is G/C.
