# Supplementary material for: COVID-19: molecular and serological detection methods
Source: PeerJ. 2020 Oct 7;8:e10180. doi: 10.7717/peerj.10180 (PMC7547594; doi:10.7717/peerj.10180)
Supplement: Supplemental Information 2 [file peerj-08-10180-s002.docx]

**Table S2:**

**The rRT-PCR controls with expected results and interpretations**

| **rRT-PCR**  **control name** | **rRT-PCR**  **control type** | **Expected cq**  **values** | **N1** | **N2** | **RP** | **Interpretation** |
| --- | --- | --- | --- | --- | --- | --- |
| nCoVPC | Positive | <40 | + | + | + | Reagent failure (e.g. primer and probe integrity |
| NTC | Negative | None detected | - | - | - | Reagent and/or environmental contamination |
| HSC | Extraction | <40 | - | - | + | Extraction issues |
